# Training-Induced Muscle Adaptations During Competitive Preparation in Elite Female Rowers

**DOI:** 10.3389/fspor.2021.781942

**Published:** 2021-12-08

**Authors:** Stephan van der Zwaard, Tommie F. P. Koppens, Guido Weide, Koen Levels, Mathijs J. Hofmijster, Jos J. de Koning, Richard T. Jaspers

**Affiliations:** ^1^Department of Human Movement Sciences, Amsterdam Movement Sciences, Vrije Universiteit Amsterdam, Amsterdam, Netherlands; ^2^Laboratory for Myology, Department of Human Movement Sciences, Amsterdam Movement Sciences, Vrije Universiteit Amsterdam, Amsterdam, Netherlands; ^3^Leiden Institute of Advanced Computer Science, Leiden University, Leiden, Netherlands; ^4^Royal Dutch Rowing Federation, Amstelveen, Netherlands; ^5^Faculty of Sports and Nutrition, Amsterdam University of Applied Sciences, Amsterdam, Netherlands

**Keywords:** rowing, skeletal muscle adaptation, hypertrophy, muscle architecture, fascicle length, 3-D ultrasound imaging

## Abstract

Training-induced adaptations in muscle morphology, including their magnitude and individual variation, remain relatively unknown in elite athletes. We reported changes in rowing performance and muscle morphology during the general and competitive preparation phases in elite rowers. Nineteen female rowers completed 8 weeks of general preparation, including concurrent endurance and high-load resistance training (HLRT). Seven rowers were monitored during a subsequent 16 weeks of competitive preparation, including concurrent endurance and resistance training with additional plyometric loading (APL). *Vastus lateralis* muscle volume, physiological cross-sectional area (PCSA), fascicle length, and pennation angle were measured using 3D ultrasonography. Rowing ergometer power output was measured as mean power in the final 4 minutes of an incremental test. Rowing ergometer power output improved during general preparation [+2 ± 2%, effect size (ES) = 0.22, *P* = 0.004], while fascicle length decreased (−5 ± 8%, ES = −0.47, *P* = 0.020). Rowing power output further improved during competitive preparation (+5 ± 3%, ES = 0.52, *P* = 0.010). Here, morphological adaptations were not significant, but demonstrated large ESs for fascicle length (+13 ± 19%, ES = 0.93), medium for pennation angle (−9 ± 15%, ES = −0.71), and small for muscle volume (+8 ± 13%, ES = 0.32). Importantly, rowers showed large individual differences in their training-induced muscle adaptations. In conclusion, *vastus lateralis* muscles of elite female athletes are highly adaptive to specific training stimuli, and adaptations largely differ between individual athletes. Therefore, coaches are encouraged to closely monitor their athletes' individual (muscle) adaptations to better evaluate the effectiveness of their training programs and finetune them to the athlete's individual needs.

## Introduction

Training-induced adaptations are essential for elite athletes to optimize their performance in preparation to the World Championships or the Olympics. Adaptations are known to be specific to the applied training stimulus, which can be described in terms of volume, intensity, frequency, joint range of motion and mode of resistance and/or endurance training (Coffey and Hawley, 2017). Important training adaptations are observed in the skeletal muscle. Recent studies have examined how such skeletal muscle characteristics are linked to the performance of athletes (van der Zwaard et al., 2018a,b). These studies show that muscle volume and architecture are important determinants for sprint and endurance performance of elite rowers and cyclists (van der Zwaard et al., 2018a,b). Muscle volume of the *vastus lateralis*, for example, largely explains differences in rowing ergometer performance in Olympic male and female rowers (van der Zwaard et al., 2018b). In addition, male cyclists with a large physiological cross-sectional area (PCSA) of the *vastus lateralis* demonstrate a relatively low endurance performance, whereas cyclists with longer muscle fascicles show better combined sprint and endurance performance (van der Zwaard et al., 2018a). Insight into muscle adaptations that result from specific training interventions may therefore deepen our understanding of how athletes can optimize their performance.

Resistance training plays an important role in the training program of rowers (Lawton et al., 2011). High-load resistance training (HLRT) is widely practiced and is known to increase muscle volume by muscle fiber hypertrophy and to increase pennation angle (Folland and Williams, 2007; Timmins et al., 2016). Recently, a review of the literature revealed that plyometric training, which involves a sequence of rapid eccentric and concentric actions, may also induce whole-muscle hypertrophy, at least for the lower extremity muscles (Grgic et al., 2020). Moreover, plyometric training likely contributes to an increase in fascicle length, similar to other eccentric or eccentrically-based training approaches (Timmins et al., 2016). Training effects of both HLRT and plyometric training have been studied extensively (Folland and Williams, 2007; Ramirez-Campillo et al., 2018); however, most studies focus on untrained or recreationally trained participants rather than on elite athletes (Folland and Williams, 2007; Grgic et al., 2020). In general, skeletal muscle adaptations following training are considered to be marginal in highly-trained athletes, especially when resistance training is concurrently performed with endurance training (Coffey and Hawley, 2017). However, large differences in skeletal muscle adaptations may exist between individuals, even in elite athletes (Hubal et al., 2005). To the best of our knowledge, individual adaptations in morphology of the quadriceps muscle in response to competitive preparation consisting of concurrent endurance and heavy resistance or plyometric resistance training have not been investigated in elite rowers.

The aim of this study was to investigate morphological and functional adaptations during the general and competitive preparation phases in elite female rowers. The study focuses on muscle morphology of the *m. vastus lateralis*, one of the quadriceps muscles, which is essential to the power production in rowing (Guével et al., 2011) and is the most superficial and largest of the *quadriceps femoris* muscles in both rowers and controls (Ema et al., 2014). We hypothesized an increase in PCSA after the general preparation that consisted of concurrent endurance and HLRT. With additional plyometric loading (APL), we expected an increase in fascicle length during the competitive preparation that included concurrent endurance and HLRT. Finally, we hypothesized that on average, adaptations in performance and muscle morphology after the general and competitive preparation will be small in these female elite athletes, but that considerable differences exist between individuals.

## Methods and Materials

### Participants

Twenty-one elite female rowers volunteered to participate in this study (age 26 ± 4 years; height 1.81 ± 0.06 m; weight 74.2 ± 7.3 kg). All rowers were professional athletes of the national team, had 5.9 ± 2.2 years of rowing experience, and trained for at least 12 sessions each week. Participants included 19 heavyweight and two lightweight rowers. All rowers competed in international rowing regattas, 19 participated in Rowing World Championships, and 11 participated in the Olympic Games. Five participants won Olympic medals. Participants completed a 2,000-m ergometer test in 414.2 ± 10.8 s (range: 399.3–437.8 s), placing them between the 95th and 99th percentiles of the international ranking for indoor rowing in 2019 (Concept II) (Concept2 Logbook, 2019) | 2,000m Indoor Rower Rankings). Prior to participation, participants were informed about the experimental procedures of the study and provided written informed consent. The study was conducted according to the principles of the Declaration of Helsinki and was approved by the departmental ethics committee of the Vrije Universiteit, Amsterdam, the Netherlands (VCWE-2020-122).

### Experimental Design and Training Regimens

This study was an observational non-randomized training study, in which all rowers were monitored during their general preparation phase and a subgroup of seven rowers were monitored during their competitive preparation phase (see Table 1). Testing was performed and muscle morphology was assessed the week before (pre) and in the week after general preparation (post-1) and in the week after competitive preparation in a subgroup of seven female rowers (post-2). Both training regimens were completed during preseason, when athletes prepared for the upcoming competitive season. For reasons unrelated to the study, two participants did not complete the post-1 measurement and were excluded from analysis.

**Table 1:** Training distribution during general preparation and competitive preparation in elite female rowers [training sessions per training (sub)type and total duration of training per week].

**Training type**	**General preparation (8 weeks)**	**Competitive preparation (16 weeks)**
Endurance training (boat or rowing/cycle ergometer)	10	11
Race intensity	1%	0%
Medium intensity	10%	13%
Anaerobic threshold	13%	11%
Steady state	35%	33%
Steady state + sprints	0%	4%
Steady state + accelerations	1%	6%
Slow steady state	32%	29%
Power endurance	8%	3%
Core-Stability training	2	2
High-load resistance training	2	1
Additional plyometric loading	–	1
Total duration	16–20 h	16–24 h

During the general preparation, participants performed 8 weeks of concurrent training with emphasis on HLRT. They performed two HLRT sessions per week, with the aim to increase muscle force production by increasing the PCSA of their muscles. Participants executed resistance exercises with high load, aiming for 3–4 sets of 5–10 repetitions per set with an intensity of ~70–85% of the 1-repetition maximum. HLRT sessions included exercises such as deadlifts, squats, bench press, and unilateral leg exercises, which were all typical exercises performed by the participants in their previous resistance training program. During their competitive preparation, a subgroup of seven female rowers completed a subsequent 16 weeks of concurrent endurance and HLRT with APL, aiming to increase muscle power and longitudinal growth of their muscle fibers. In addition to a session of regular resistance training, rowers executed one session of APL per week, which consists of exercises such as counter movement jumps, aqua bag squat jumps, dumbbell broad jumps, and stretch exercises. Participants were instructed to execute these exercises in circuit, using a large range of motion and with a high velocity. Each circuit consisted of 4–6 exercises and exercises were executed with 3–5 sets of 2–10 repetitions. Note that participants performed both HLRT and APL sessions in addition to their other training, which consisted of core stability (two times a week) and row-specific endurance training sessions (8–12 times a week, see Table 1). For weekly details of the resistance training sessions, see Supplementary Table 1. Training sessions were performed on-site at the facilities of the Royal Rowing Federation and were overseen by the resistance and conditioning coach of the female rowing team.

### Methodology

#### 3-Dimensional Ultrasound

Muscle morphology of the *vastus lateralis* was obtained using 3D ultrasound imaging, as described previously (Weide et al., 2017; van der Zwaard et al., 2018b). Images were acquired from the participant's right leg using a 5-cm linear probe (Technos MPX, ESAOTE S.p.A. Italy) in B-mode (25 Hz), while she was positioned on a bench with a hip flexion angle of 85° and knee flexion angle of 60°. To prevent displacement or rotation of the legs, the lower leg was strapped and secured to the bench. Ultrasound sweeps of the *m. vastus lateralis* were collected in longitudinal direction, starting from the lateral border of the muscle and scanning from the distal to proximal end of the muscle with the probe in transverse orientation. Subsequent sweeps were taken medially from the previous sweep with a 1-cm overlap. Location and orientation of the ultrasound probe were collected over time using a cluster marker that was attached to the ultrasound probe and using a motion capture system (Optotrak Certus; Northern Digital, Waterloo, ON, Canada). An external trigger marked the start of each scan and was used to synchronize position data with the ultrasound B-mode images to enable construction of a 3D voxel array using customized software (MatLab; MathWorks, Natick, MA, USA) (Weide et al., 2017).

The ultrasound voxel array was then analyzed using 3D image-processing software (MITK; http://www.mitk.org/) (Weide et al., 2017). Firstly, the origin and distal end of the muscle belly of the *m. vastus lateralis* were identified. Secondly, fascicle length and pennation angle (the angle between fascicle and distal aponeurosis) were obtained from the mid-longitudinal “fascicle” plane, at 2/3rd of the muscle belly and distal to the trochanter major (van der Zwaard et al., 2018b). Finally, muscle volume was derived from manual segmentation of anatomic cross-sections along the length of the *vastus lateralis*, between the distal and proximal end of the muscle belly. Adipose tissue and connective tissue incursions were carefully excluded from segmentation, and interactive interpolation was performed to calculate muscle volume from the 3D ultrasound voxel array. PCSA was calculated as muscle volume divided by fascicle length.

Test–retest reliability of the 3D ultrasound measurements has been established for our lab using intraclass correlation (ICC) and standard error of measurement (SEM). ICC was calculated with a two-way mixed model and absolute agreement using “single measures” for volume and “average measures” for PCSA, pennation angle, and fascicle length (mean of three measurements). ICC values were rated as excellent, with the following values for muscle volume = 0.987, PCSA = 0.984, pennation angle = 0.953, and fascicle length = 0.959. The minimal detectable change (MDC) was then calculated based on these ICC values and the standard deviation of baseline values, using the following formula: MDC = SD × √(1-ICC) × 1.96 × √2 (Weir, 2005). This MDC values, which indicate the minimal increase or decrease that would represent a real change, were the following values for muscle volume (±32.5 mL), PCSA (±2.91 cm^2^), pennation angle (±1.32°), and fascicle length (±0.64 cm).

#### Rowing Ergometer Performance

Maximal power output was obtained from a maximal incremental test to voluntary exhaustion. The test was conducted on a Concept II ergometer (Concept II, Model D, Morrisville, VT, USA), to avoid confounding environmental effects (e.g., wind or water current), and consisted of seven 4-min stages with one min of rest between each stage. Rowers started at a power output corresponding to 40% of their estimated 2,000-m mean power, and power output was increased by 8% for every subsequent 4-min stage (Rice, 2011). The final 4-min exercise stage was an all-out block. The produced power output was averaged over this final stage to obtain the average maximal power output, which is considered to be an important predictor of rowing ergometer performance (Bourdin et al., 2004). Our (unpublished) data showed that these maximal power values were strongly related to the 2,000-m rowing ergometer finish times in our subjects (*r* = −0.95, data not shown). Participants were instructed to avoid strenuous exercise the day before each rowing ergometer test. Due to reasons unrelated to the study (e.g., sickness), two female rowers were excluded from analysis of rowing ergometer power output, as either pre- or post-1 measurements could not be obtained in these rowers.

### Statistical Analysis

Data are presented as individual values or as mean ± SD. Paired-sampled *t*-tests or, when data were not normally distributed, non-parametric Wilcoxon signed-rank tests were used to detect differences in morphological and functional adaptations after general preparation (pre–post-1) and after competitive preparation in a subgroup of rowers (post-1–post-2). Magnitude of the effects was assessed by effect size (ES) that were obtained as the mean difference divided by the pooled standard deviation. ESs were reported in standardized Cohen's *d* units with their 95% confidence interval, and absolute ESs were considered to be negligible (<0.20), small (0.20–0.50), medium (0.50–0.80), or large (>0.80) (Cohen, 1992). Individual training adaptations were reported, and relationships between individual adaptations in PCSA and fascicle length (expressed relative to baseline) were assessed using Pearson or Spearman correlations. Stepwise multiple regression analyses were performed to explain changes in rowing ergometer power output based on baseline values and changes in muscle morphology. Differences were considered statistically significant if *p* < 0.05. Tendencies were reported for *p* < 0.10.

## Results

### Training Adaptations During the General Preparation Phase

**Figure 1:**
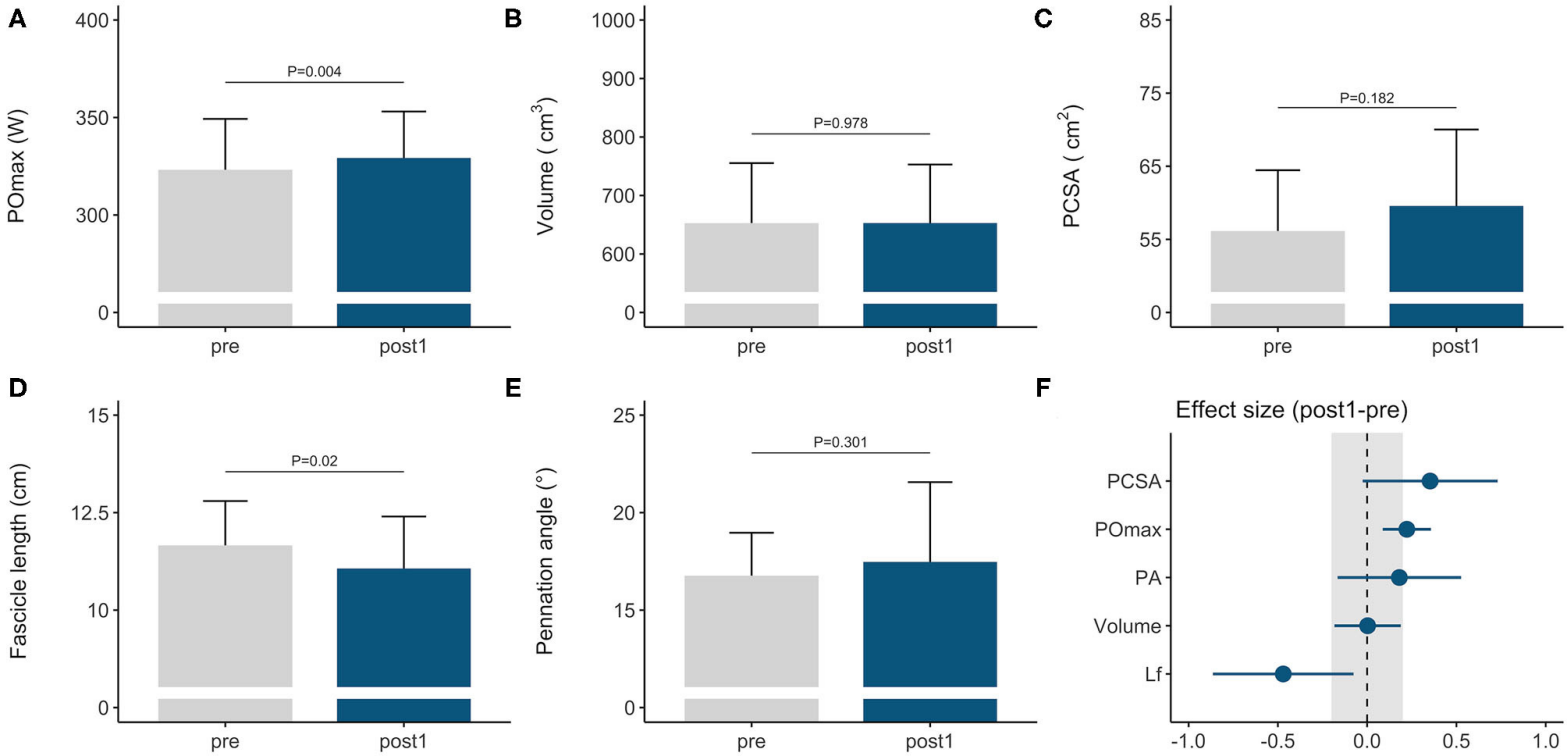
Morphological and functional responses to the general preparation phase in elite female rowers. Functional response is shown for **(A)** maximal power output obtained on a rowing ergometer during concurrent endurance and high-load resistance training. Morphological responses are shown for *m. vastus lateralis* **(B)** volume, **(C)** PCSA, **(D)** fascicle length, and **(E)** pennation angle. Standardized ESs and their 95% confidence intervals (in Cohen's *d*) are reported in **(F)** for the pre–post differences. The gray area in **(F)** highlights the smallest worthwhile change (|ES| = 0.2), illustrating what effects can considered to be non-trivial. PCSA, physiological cross-sectional area; POmax, rowing ergometer power output in the final 4 min of a maximal incremental test; Lf, fascicle length.

Rowers improved their rowing ergometer power output by 2 ± 2% during the general preparation phase, consisting of concurrent endurance and HLRT (Table 2, Figure 1A). Rowing ergometer power output per kg body mass showed similar improvement (+2 ± 3%, ES = 0.42, *P* = 0.002), as no changes in body mass were observed (−0.32 ± 0.85 kg, *P* > 0.05, ES = −0.04). Morphological adaptations included changes in *vastus lateralis* muscle architecture, with no change in muscle volume and a small reduction in fascicle length (Table 2, Figures 1B–E). Effect sizes of training adaptations have been summarized in Figure 1F. Stepwise multiple regression analysis revealed that changes in rowing ergometer power output were largely explained by the baseline values of muscle volume, pennation angle, and power output (*R*^2^ = 0.68, *P* < 0.01). When baseline power output was excluded as a predictor, the change in rowing ergometer power output tended to be explained by the changes in muscle volume and baseline values of pennation angle (*R*^2^ = 0.32, *P* = 0.068).

**Table 2:** Functional and morphological adaptations following the general preparation phase and competitive preparation phase in elite female rowers.

**Training period**	**Parameter**	**Change**	**Change (%)**	**Effect size (Cohen's d)**		***P*-Value**
General preparation phase (*n* = 19)	Rowing ergometer performance	+5.9 ± 7 W	+2 ± 2%	0.22	Small	0.004^*^
	Muscle volume	+0.3 ± 41 mL	+0.3 ± 7%	0.003	Negligible	0.978
	Fascicle length	−0.59 ± 1.0 cm	−5 ± 8%	−0.47	Small	0.020^*^
	PCSA	+3.43 ± 8 cm^2^	+6 ± 14%	0.35	Small	0.182
	Pennation angle	+0.71 ± 3°	+4 ± 16%	0.18	Small	0.301
Competitive preparation phase (*n* = 7)	Rowing ergometer performance	+17.8 ± 11 W	+5 ± 3%	0.52	Medium	0.010^*^
	Muscle volume	+53.7 ± 84 mL	+8 ± 13%	0.32	Small	0.141
	Fascicle length	+1.21 ± 1.9 cm	+13 ± 19%	0.93	Large	0.150
	PCSA	−1.01 ± 16 cm^2^	−0.4 ± 28%	−0.07	Negligible	0.872
	Pennation angle	−1.76 ± 2°	−9 ± 15%	−0.71	Medium	0.110

* *P < 0.05*.

### Training Adaptations During the Competitive Preparation Phase

**Figure 2:**
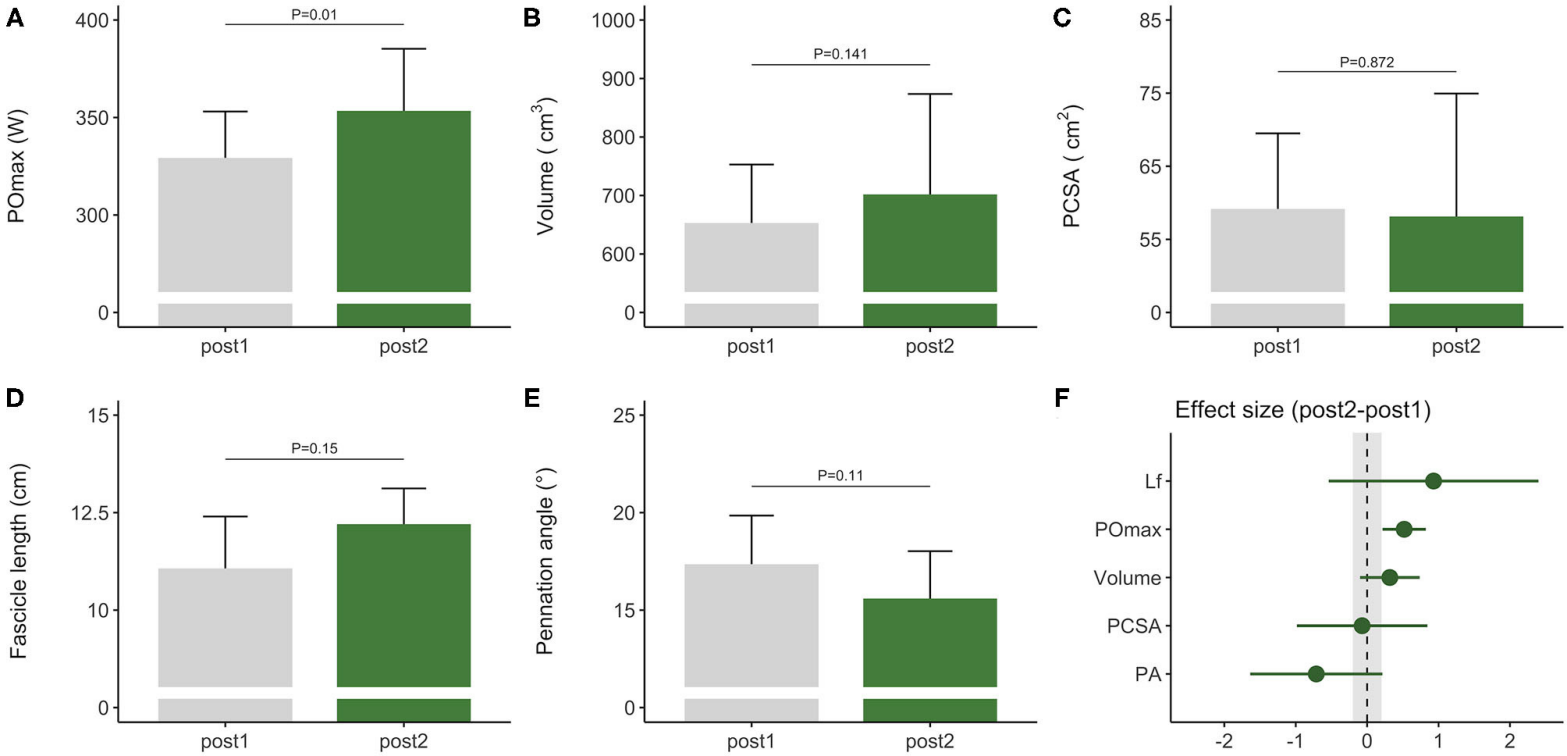
Morphological and functional responses to the competitive preparation phase in a subgroup of elite female rowers. Functional response is shown for **(A)** maximal power output obtained on a rowing ergometer during concurrent training with additional plyometric loading. Morphological responses are shown for *m. vastus lateralis* **(B)** volume, **(C)** PCSA, **(D)** fascicle length, and **(E)** pennation angle. Standardized ESs and their 95% confidence intervals (in Cohen's *d*) are reported in **(F)** for the pre–post differences. The gray area in **(F)** highlights the smallest worthwhile change (|ES| = 0.2), illustrating what effects can considered to be non-trivial. PCSA, physiological cross-sectional area; POmax, rowing ergometer power output in the final 4 min of a maximal incremental test; Lf, fascicle length.

During the competitive preparation phase, a subgroup of participants further improved their rowing ergometer power output by 5 ± 3% following concurrent endurance and HLRT with APL (Table 2, Figure 2A). Similarly, rowing ergometer power output per kg body mass showed a tendency to improve (+6 ± 5%, ES = 0.74, *P* = 0.063), since no changes in body mass were observed (−0.35 ± 1.63 kg, *P* > 0.05, ES = −0.03). Morphological adaptations were non-significant in this small sample of seven rowers (Table 2, Figures 2B–E), but average ES was large for fascicle length, medium for pennation angle, small for muscle volume, and negligible for PCSA (Table 2). Effect sizes of training adaptations have been summarized in Figure 2F. Data were insufficient (*n* = 6) to model changes in rowing ergometer power output using stepwise multiple regression analyses. In brief, results might indicate large but non-significant increases in fascicle length, and no changes in PCSA.

### Individualized Training Adaptations

**Figure 3:**
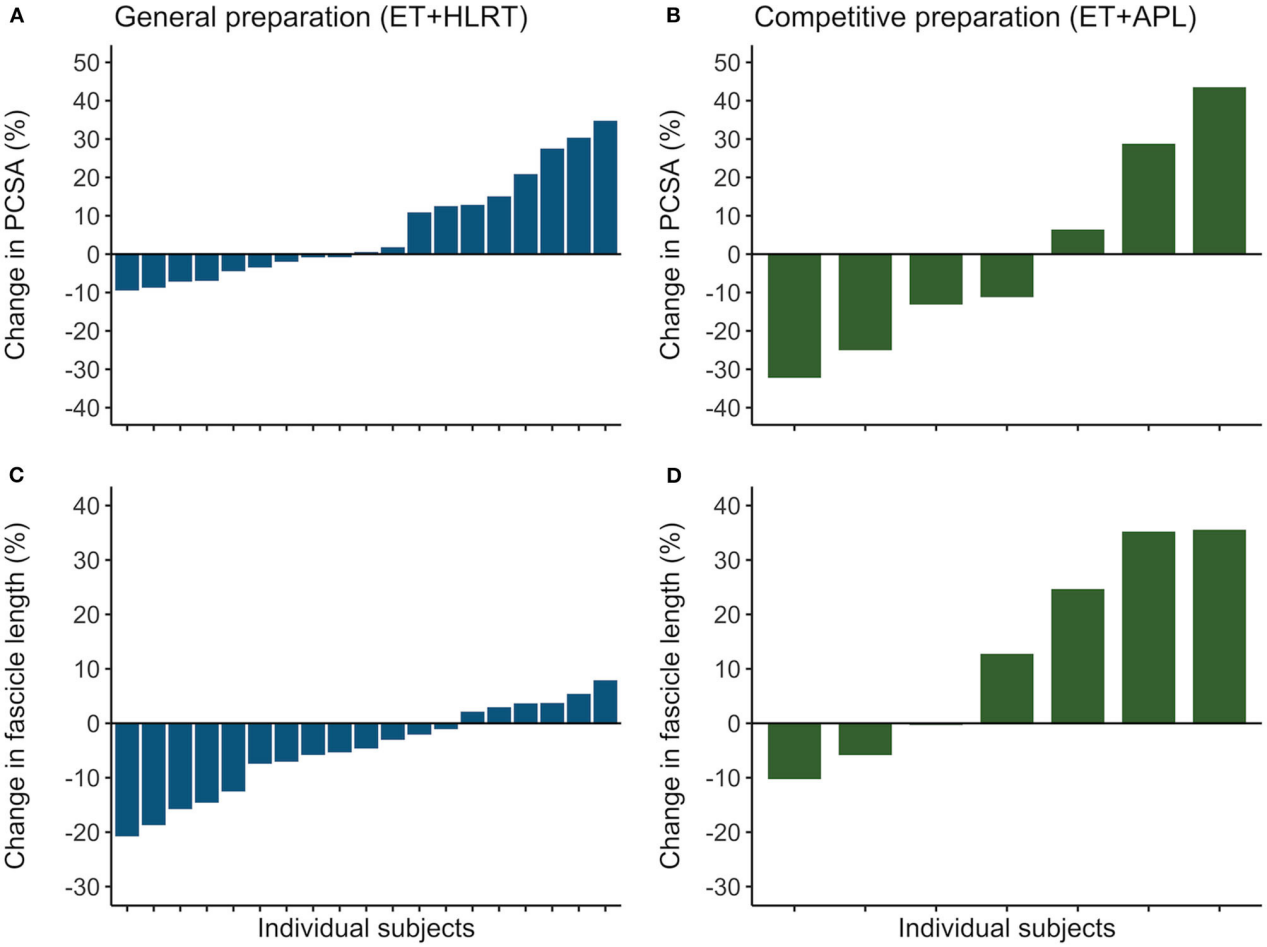
Individual training adaptations during the general and competitive preparation phases in elite female rowers. Percentual changes in *vastus lateralis* **(A)** PCSA and **(C)** fascicle length are shown for the individual rowers during the general preparation phase. In a subgroup of rowers, percentual changes are reported for **(B)** PCSA and **(D)** fascicle length during the competitive preparation phase. ET, endurance training; HLRT, high-load resistance training; APL, high-load resistance training with additional plyometric loading.

Rowers showed substantial differences in their individual adaptations in the general preparation phase for muscle volume (ranging from −10 to +15%), fascicle length (−21 to +8%), and PCSA (−9 to +35%). Also, the subgroup of rowers revealed different individual adaptations after competitive preparation for muscle volume (−8 to +29%,), fascicle length (−10 to +36%), and PCSA (−32 to +44%). Similarly, confidence intervals of the ESs indicated large individual differences following the general preparation phase for muscle volume (ES from −0.18 to 0.19), fascicle length (−0.86 to −0.08), and PCSA (−0.03 to 0.73) and following the competitive preparation phase for muscle volume (−0.10 to 0.74), fascicle length (−0.54 to 2.40), and PCSA (−0.99 to 0.84). In Figure 3, the individualized training adaptations are shown for fascicle length and PCSA after the general and competitive preparation phases. Results illustrate large adaptations and large individual differences in training adaptations of *vastus lateralis* muscle volume and architecture in elite athletes.

### Relationships Between Muscle Hypertrophy and Fascicle Growth

**Figure 4:**
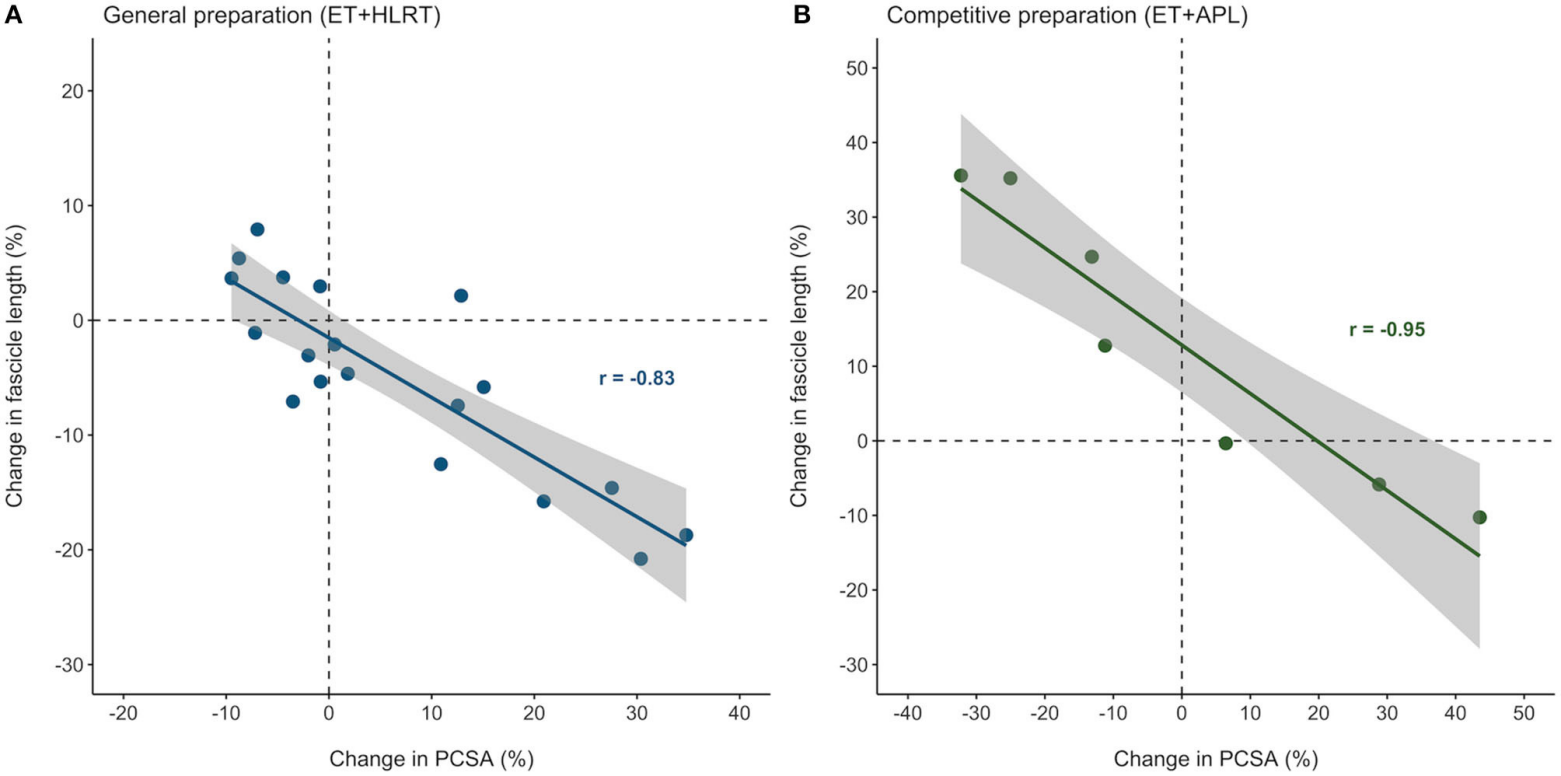
Individual adaptations in muscle architecture were strongly related in elite female rowers, after both the general and competitive preparation phases. Percentual changes in *vastus lateralis* PCSA and fascicle length were strongly related during **(A)** the general preparation phase and **(B)** the competitive preparation phase. ET, endurance training; HLRT, high-load resistance training; APL, high-load resistance training with additional plyometric loading.

Figure 4 illustrates that individual adaptations in fascicle length and PCSA were strongly and inversely related, after both the general preparation and competitive preparation (*r* = −0.83, *P* < 0.01 and *r* = −0.95, *P* < 0.01, respectively). These results indicate that even though individual training adaptations may differ, changes in muscle architecture of the *m*. *vastus lateralis* are highly interrelated, both following concurrent training with a focus on HLRT and concurrent training with APL.

## Discussion

The aim of this study was to investigate morphological and functional responses to general and competitive preparation in elite female rowers. Participants improved their rowing ergometer power output, during both the general and competitive preparation phases. General preparation (focused on HLRT) revealed no change in volume, surprisingly no muscle hypertrophy, and a small decrease in fascicle length. During the competitive preparation phase (including APL), a subgroup of seven rowers demonstrated large but non-significant increases in fascicle length, and no changes in PCSA. Average adaptations in rowing ergometer power output and muscle morphology were larger than expected for this group of elite athletes, particularly during the competitive preparation phase. Moreover, we observed large individual differences in training-induced adaptations in their *vastus lateralis* muscle volume and architecture.

### Muscle Morphology in Rowers

Although many studies have investigated the contribution of muscle strength, power, muscle activation, kinematics, or muscle fiber composition to rowing performance (Secher, 1983; Hagerman, 1984; Hofmijster et al., 2007; Lawton et al., 2011; Miarka et al., 2018; Volianitis et al., 2020), the literature on muscle morphology in rowers remains limited. Initially, studies focused on anatomical cross-sectional area (ACSA) of the quadriceps femoris, demonstrating that mid-thigh ACSA in elderly oarsman was larger compared to that of sedentary controls, and significantly related to 2000-m ergometer performance (Yoshiga et al., 2002) and to leg extension power (Asaka et al., 2010). ACSA of the *m. vastus lateralis* was also larger in young rowers compared to that of controls over the entire muscle length [at 30, 50, and 70%; (Ema et al., 2014)]. These authors also demonstrated that *m. vastus lateralis* is the largest of the *quadriceps femoris* muscles, and that the *m. vastus lateralis* comprises a larger percentage of the total *quadriceps femoris* volume in rowers compared to controls (Ema et al., 2014). To the best of our knowledge, there is one previous study (van der Zwaard et al., 2018b) that reported muscle morphology in terms of physiological CSA, fascicle length, pennation angle, and also muscle volume of the *vastus lateralis* in elite rowers. Results showed that *m. vastus lateralis* volume was strongly related to 2,000-m rowing ergometer performance and that its muscle architecture impacted lower body peak power (van der Zwaard et al., 2018b). In this study, muscle volume at baseline (653 ± 103 mL) was similar to that previously reported in Olympic female rowers (van der Zwaard et al., 2018b). Surprisingly, rowers in the present study displayed smaller PCSA (56.1 ± 8.3 cm^2^) and pennation angles (16.8 ± 2.2°), and larger fascicle lengths (11.7 ± 1.1 cm) compared to this previous study (van der Zwaard et al., 2018b), possibly due to differences in their training history. To the authors' knowledge, our study is the first presenting training-induced adaptations of whole-muscle morphology of *m. vastus lateralis* and performance during the general and competitive preparation in elite rowers.

### Training-Induced Adaptations in Muscle Morphology and Performance

Rowing ergometer power output increased by 2% during the general preparation phase, consisting of 8 weeks of concurrent training with a focus on HLRT. In a subgroup of rowers, power output further improved by 5% after competitive preparation, consisting of 16 weeks of concurrent training with a focus on APL. In a recent meta-analysis, Thiele et al. (2020) showed that recreational and subelite level rowers improved their sport-specific rowing performance (e.g., 2,000-m rowing ergometer time) following 4–9 weeks of resistance training with a small effect (ES = 0.32). Similar or even larger effect sizes were observed in elite female rowers of our study for improvements in maximal rowing ergometer power output, which is a critical determinant of 2,000-m rowing ergometer performance (Bourdin et al., 2004), during the general (ES = 0.22) and competitive preparation phases (ES = 0.52). Contrary to our expectations, these results illustrate that compared to resistance training in recreational or subelite rowers, elite rowers demonstrate similar or even larger improvements of their rowing ergometer power output after concurrent resistance and endurance training.

Muscle volume remained unchanged during the general preparation phase with concurrent training focused on HLRT, whereas *vastus lateralis* fascicle length decreased (−5 ± 8%, ES = −0.47). Surprisingly, PCSA did not change, which was likely due to the large individual differences in muscle hypertrophy (+6 ± 14%, ES = 0.35). HLRT is known to increase muscle size (ACSA, PCSA, and muscle volume) after 8–14 weeks of training, with muscle fiber hypertrophy as primary underlying adaptation (Abe et al., 2000; Aagaard et al., 2001; Folland and Williams, 2007). Muscle fiber hypertrophy is observed in all fiber types following 8 weeks of heavy resistance training, both in men and in women (Staron et al., 1994). Furthermore, men and women show similar relative improvements in hypertrophy following lower body HLRT (Abe et al., 2000; Folland and Williams, 2007). Recently, it was observed that resistance training with high loads induced similar whole-muscle hypertrophy (+8.3% increase, ES = 0.53) compared to training with low loads [+7.0% increase, ES = 0.42; (Schoenfeld et al., 2017)]. Although these adaptations are slightly larger than the ES we observed for PCSA (+6%, ES = 0.35), this was not unexpected, as this meta-analysis predominantly included studies with untrained individuals (Schoenfeld et al., 2017). It remains unknown whether training with high vs. low loads will evoke similar adaptations in elite athletes and whether training regimens may lead to different adaptations at the muscle fiber level, such as type II muscle hypertrophy with HLRT and type I muscle fiber hypertrophy in training with low loads (Grgic and Schoenfeld, 2018). In addition, our results show that *vastus lateralis* fascicle length decreased during 8 weeks of concurrent training with HLRT (−5 ± 8%, ES = −0.47), contrasting previous observations with 10 weeks of concentric HLRT in recreationally active participants [+4–7%; (Blazevich et al., 2007; Franchi et al., 2014; Trezise and Blazevich, 2019)]. Although this explains why muscle volume was not increased in elite rowers of our study, further investigation should elucidate whether this adaptation in fascicle length is distinct for elite athletes. Potentially, the number of sarcomeres in series is reduced to accommodate a shift in optimum knee angle toward extension, such that sarcomere optimum length on the active length-tension curve occurs at optimum joint angle (Herring et al., 1984; Bobbert et al., 2020). In brief, here we show that 8 weeks of general preparation (concurrent training focused on HLRT) did not change muscle volume but reduced fascicle length and resulted in a 2% improvement in rowing ergometer power output.

A subgroup of rowers showed small but non-significant increases in muscle volume after competitive preparation consisting of concurrent training focused on APL, together with large but non-significant increases in fascicle length (+13 ± 19%, ES = 0.93) and no changes in PCSA. The large average ES for fascicle length likely relates to stretching of fascicles that occurs during the rapid eccentric contractions in the stretch-shortening cycles involved in the APL (Grgic et al., 2020; Monti et al., 2020). Yet, morphological adaptations did not reach statistical significance, likely due to the small sample (*n* = 7). Previous literature showed that fascicle length increased rapidly after 5 weeks of eccentric training in humans (+3%), with no further increases at 10 weeks (Blazevich et al., 2007). In young men, such increases in fascicle length have still been observed after 10 weeks of eccentric training [+12%; (Franchi et al., 2014)]. Few studies have examined muscle architecture following plyometric training. However, recently, time course changes in fascicle length with plyometric training were studied in healthy men (Monti et al., 2020). It was shown that fascicle length changes could be observed already after 2 weeks including six sessions of plyometric training (+2.2%), with further increases after 4 (+4%) and 6 weeks [+4.4%; (Monti et al., 2020)]. Our results show relatively large but nonsignificant increases in fascicle length (+13%) after 16 weeks of concurrent training with APL, similar to the changes observed with 10 weeks of accentuated eccentric loading in resistance-trained men [+14%; (Walker et al., 2020)]. It remains to be determined whether the time course of fascicle length adaptations in elite athletes is similar to that of healthy men. A recent meta-analysis indicated that plyometric and resistance training interventions may produce similar effects on whole-muscle hypertrophy in untrained individuals (Grgic et al., 2020). However, in this study, PCSA did not change with concurrent training focused on APL (ES = −0.07), illustrating that radial muscle hypertrophy was negligible in elite athletes. Similarly, it was previously observed that pennation angle increased in untrained individuals with plyometric [+6%; (Monti et al., 2020)] and eccentric training +5–21%; (Blazevich et al., 2007; Franchi et al., 2014)], however, pennation angle decreased in the rowers in our study after concurrent training with APL (−2°, −9 ± 15%, ES = −0.71), similar to observations in elite athletes after sprint and jump training [−3°; (Blazevich et al., 2003)]. Therefore, adaptations in muscle morphology are likely different in elite athletes compared to untrained individuals. Our findings show that 16 weeks of competitive preparation (concurrent training with APL) may have induced longitudinal fascicle growth instead of radial hypertrophy. Also, rowing ergometer power output was improved by 5 ± 3%.

Adaptations in fascicle length could have mechanical and metabolic implications. Changes in fascicle length underpin changes in torque–angle relationships (Blazevich et al., 2007) and attribute to a shift in optimum knee angle toward flexion in simulated isokinetic sprint cycling (Bobbert et al., 2020). Here, longer fascicles also shift optimal power production toward higher pedaling rates and interestingly lead to higher peak power production (Bobbert et al., 2020). This seems to be explained by a better alignment with the peak power vs. pedaling rate curves of the other leg muscles (Bobbert et al., 2020). The same may apply to rowers, who aim to produce high power outputs while attaining high stroke frequencies. Importantly, long fascicles are also advantageous for athletes that need to combine a well-developed sprint and endurance performance (van der Zwaard et al., 2018a,b), such as rowers. Likely, this is because longer fascicles, in contrast to a larger PCSA, do not negatively affect the diffusion distance of the muscle fibers, hampering the balance between oxygen supply and demand in the muscle (van der Zwaard et al., 2018a). In the late 2000s, it has been suggested that adaptations in fascicle length are most likely influenced by training range of motion rather than contraction velocity or contraction mode (Blazevich et al., 2007). Recently, it was confirmed that longitudinal fascicle growth seems to be independent of lengthening velocity (Marzilger et al., 2020). For contraction mode, however, eccentric and concentric training show distinct differences in adaptations of fascicle length when training load is matched for their concentric or eccentric 1-repetition maximum [+12 vs. +5%; (Franchi et al., 2014)]. Mechanical stretch is likely an essential stimulus for longitudinal fascicle growth, but may be less prominent in plyometric training compared to the stretch observed with eccentric training (Monti et al., 2020). We suggest future studies to investigate the time course and critical stimuli for training-induced longitudinal and radial muscle growth, also in elite athletes.

### Individual Differences in Training Adaptations of Muscle Morphology

In elite female rowers, we demonstrated considerable individual differences in training adaptations of *vastus lateralis* muscle volume and architecture during general and competitive preparation. To the authors' knowledge, only one study exists presenting individual differences in fascicle length adaptations in resistance-trained men (Walker et al., 2020). These authors observed that changes in fascicle length differed considerably between participants after traditional resistance training (~−21 to +16%) and accentuated eccentric loading [~−7 to +31%; (Walker et al., 2020)], reporting similar ranges compared to those of the elite rowers in our study after concurrent training with HLRT (−21 to +8%) and concurrent training with APL (−10 to +36%), respectively. In addition, this study also demonstrates that training-induced adaptations in muscle morphology can largely differ between individual female rowers, following general and also competitive preparation. Overall, the ESs for adaptations in muscle morphology ranged from largely negative to largely positive (−0.99 to 2.40). Interestingly, we also discovered strong negative relationships between percentual changes in PCSA and fascicle length, which were observed based on individual adaptations both after general (*r* = −0.83) and competitive preparation (*r* = −0.95). These results contribute to our understanding of individualized training adaptations in elite female athletes and highlight that similar training stimuli may evoke remarkable differences in training-induced morphological adaptations, even in elite athletes. Therefore, researchers and coaches are encouraged to finetune and monitor training progression of the individual athletes and investigate the specificity of individual training adaptations in more detail.

### A Case for Monitoring Training Adaptations in Elite Athletes

Resistance and endurance training research is conducted by many exercise scientists and has a profound impact on the field of sports science (Kraemer et al., 2017; van der Zwaard et al., 2020). In this study, elite rowers were monitored during their general preparation phase consisting of concurrent training focused on HLRT and during the competitive preparation phase consisting of concurrent training with a focus on APL. Although scientists are encouraged to study the effects of (resistance) training in different populations (Kraemer et al., 2017), research on the effects of high-load resistance or plyometric training in elite athletes remains limited (Schoenfeld et al., 2017; Grgic et al., 2020). In rowing, a recent meta-analysis (Thiele et al., 2020) revealed only two studies that investigated resistance training in elite rowers (Lawton et al., 2012, 2013). However, training-induced changes in muscle morphology of elite rowers have not yet been reported. Our study shows that during their (competitive) preparation, elite athletes are able to largely alter their muscle morphology, while improving their rowing ergometer power output. The magnitudes of training-induced changes in PCSA after general preparation and in fascicle length after competitive preparation in the female athletes in our study were shown to be remarkably similar to those previously observed after high-load resistance and plyometric training in untrained and trained men (Franchi et al., 2014; Schoenfeld et al., 2017; Walker et al., 2020), which is in line with previous suggestions (Folland and Williams, 2007). However, unlike untrained individuals, these changes came at the cost of a lower fascicle length and a decreased pennation angle, respectively, suggesting that adaptations in muscle morphology may be more tightly regulated in elite athletes.

### Limitations

It is commonly acknowledged that insight into training adaptations of elite athletes is largely built upon empiric studies, which is an inherent limitation of performing experimental training studies with elite athletes (Volianitis et al., 2020). This study is no exception in this respect, with athletes performing concurrent training regimens during their general and competitive preparation. While our study was performed under highly ecologically valid conditions, the experimental design of the study did not allow for inclusion of a control group. Therefore, we cannot rule out that there may be other factors beyond our control that influenced the changes in muscle morphology and rowing ergometer power output in this study.

Another limitation of studying elite athletes is that there are only a few of them. Recruiting large samples of participants in training studies poses inherent difficulties already in untrained populations (Grgic et al., 2020), let alone in elite athletes. Our sample size was similar to the 10–22 athletes reported in previous training studies with elite rowers (Thiele et al., 2020). However, results of the subgroup of seven rowers who performed concurrent training with APL during the competitive preparation did not reach statistical significance, presumably because the statistical power was too low to draw inferences. This was confirmed by sample size calculations based on the current data, illustrating that sufficient statistical power was obtained for the general preparation with 19 participants (detecting morphological adaptations with ESs ≥0.35) and for the competitive preparation with 22 participants (detecting morphological adaptations with ESs ≥ 0.32). Therefore, findings support only preliminary conclusions and warrant more studies to confirm the estimation of training effects.

## Conclusion

This study shows that elite female rowers significantly improved their rowing ergometer power output by 2 ± 2 and 5 ± 3% during 8 weeks of general preparation (concurrent training focused on HLRT) and 16 weeks of competitive preparation (concurrent training with APL), respectively. Interestingly, these specific training regimens evoked a large magnitude of adaptations in *vastus lateralis* muscle morphology and also large individual differences in training adaptations in these elite female athletes. Future studies need to elucidate the regulatory mechanisms of these morphological training adaptations and differences in adaptations between individuals and populations. Coaches are advised to closely monitor their athletes' individual (muscle) adaptations using 3D ultrasound measurements, which may enable coaches to better evaluate the effectiveness of their training programs and finetune these programs to the athlete's individual needs.

## Data Availability Statement

The raw data supporting the conclusions of this article will be made available by the authors, without undue reservation.

## Ethics Statement

The studies involving human participants were reviewed and approved by the Departmental Ethics Committee of the Vrije Universiteit, Amsterdam, the Netherlands (VCWE-2020-122). The patients/participants provided their written informed consent to participate in this study. Written informed consent was obtained from the individual(s) for the publication of any potentially identifiable images or data included in this article.

## Author Contributions

GW and TK performed the research. SZ, TK, GW, and RJ analyzed the data and wrote the paper. All authors conceived and designed the research, edited and revised the manuscript, and approved the final version of the manuscript.

## Funding

This study was supported by the Netherlands Organization for Scientific Research (NWO) under Grant No. 546002012.

## Conflict of Interest

The authors declare that the research was conducted in the absence of any commercial or financial relationships that could be construed as a potential conflict of interest.

## Publisher's Note

All claims expressed in this article are solely those of the authors and do not necessarily represent those of their affiliated organizations, or those of the publisher, the editors and the reviewers. Any product that may be evaluated in this article, or claim that may be made by its manufacturer, is not guaranteed or endorsed by the publisher.
