# Supplementary material for: Training-Induced Muscle Adaptations During Competitive Preparation in Elite Female Rowers
Source: Front Sports Act Living. 2021 Dec 8;3:781942. doi: 10.3389/fspor.2021.781942 (PMC8692787; doi:10.3389/fspor.2021.781942)
Supplement: Supplementary file 1 [file Table_1.docx]

**Supplemental Table 1**. Detailed training characteristics of the resistance training sessions during the general preparation and competitive preparation in elite female rowers. For each week, the number of sets, number of repetitions and load in percentage 1-repetition maximum were given.

| **Training period** | **Week** | **Sets** | **Repetitions** | **%1-RM** |
| --- | --- | --- | --- | --- |
| General  preparation (n=19) | 1 | 3 ± 0 | 8 ± 1 | 75 ± 2 |
|  | 2 | 3 ± 0 | 11 ± 2 | 72 ± 3 |
|  | 3 | 3 ± 0 | 9 ± 2 | 72 ± 3 |
|  | 4 | 3 ± 0 | 6 ± 1 | 76 ± 1 |
|  | 5 | 4 ± 0 | 7 ± 1 | 75 ± 2 |
|  | 6 | 4 ± 0 | 7 ± 3 | 77 ± 4 |
|  | 7 | 4 ± 0 | 7 ± 4 | 73 ± 5 |
|  | 8 | 3 ± 0 | 6 ± 1 | 78 ± 1 |
|  |  |  |  |  |
| Competitive  preparation  (n=7) | 1 | 4 ± 1 | 7 ± 2 | 75 ± 4 |
|  | 2 | 3 ± 0 | 6 ± 1 | 79 ± 2 |
|  | 3 | 4 ± 1 | 6 ± 2 | 80 ± 3 |
|  | 4 | 4 ± 1 | 7 ± 3 | 83 ± 5 |
|  | 5 | 3 ± 0 | 6 ± 1 | 79 ± 2 |
|  | 6 | 4 ± 1 | 6 ± 2 | 80 ± 3 |
|  | 7 | 4 ± 1 | 7 ± 3 | 83 ± 5 |
|  | 8 | 3 ± 0 | 6 ± 1 | 74 ± 2 |
|  | 9 | 4 ± 1 | 6 ± 2 | 78 ± 3 |
|  | 10 | 4 ± 1 | 7 ± 3 | 83 ± 5 |
|  | 11 | 3 ± 0 | 6 ± 1 | 74 ± 2 |
|  | 12 | 4 ± 1 | 6 ± 2 | 77 ± 3 |
|  | 13 | 4 ± 1 | 7 ± 3 | 78 ± 4 |
|  | 14 | 3 ± 1 | 6 ± 1 | 81 ± 2 |
|  | 15 | 4 ± 1 | 6 ± 2 | 83 ± 3 |
|  | 16 | 3 ± 1 | 7 ± 2 | 72 ± 3 |
